# Adipocytokine levels mark endothelial function in normotensive individuals

**DOI:** 10.1186/1475-2840-11-103

**Published:** 2012-08-31

**Authors:** Anna Solini, Francesco Stea, Eleonora Santini, Rosa Maria Bruno, Emiliano Duranti, Stefano Taddei, Lorenzo Ghiadoni

**Affiliations:** 1 Department of Internal Medicine University of Pisa, Via Roma 67, Pisa, I-56100, Italy

**Keywords:** Endothelium, Adipocytokine, Retinol binding protein-4, Resistin

## Abstract

**Background:**

Endothelial dysfunction is an independent risk factor for cardiovascular events. Inflammatory mediators released by the adipose tissue can lead to local insulin resistance and endothelial dysfunction. This study addressed the relationship of adipocytokines with endothelial function and blood pressure.

**Methods:**

In 92 newly diagnosed, drug-naïve essential hypertensive patients (HT, mean age 49 yrs) without organ damage and 66 normotensive subjects (NT, mean age 47 yrs), by an automated system, we measured endothelium-dependent and -independent vasodilation as brachial artery flow-mediated dilation before and after administration of glyceryl-trinitrate. Retinol binding protein-4 (RBP4) and resistin levels were determined by ELISA and RIA, respectively. Oxidative stress was evaluated by measuring serum malondyaldehyde (MDA).

**Results:**

Flow-mediated dilation was significantly (p = 0.03) lower in HT (5.3 ± 2.6%) than NT (6.1 ± 3.1%), while response to glyceryl-trinitrate (7.5 ± 3.7% *vs* 7.9 ± 3.4%) was similar. RBP4 (60.6 ± 25.1 *vs* 61.3 ± 25.9 μg/ml), resistin (18.8 ± 5.3 *vs* 19.9 ± 6.1 ng/ml) and MDA levels (2.39 ± 1.26 *vs* 2.08 ± 1.17 nmol/ml) were not different in HT and NT.

RBP4 (r = −0.25; p = 0.04) and resistin levels (r = −0.29; p = 0.03) were related to flow-mediated dilation in NT, but not in HT (r = −0.03 and r = −0.10, respectively). In NT, multivariate analysis including RBP4 and confounders showed that only BMI or waist circumference remained related to flow- mediated dilation. In the multivariate model including resistin and confounders, BMI, age and resistin were significantly related to flow-mediated dilation, while only age significant correlated with this parameter when BMI was replaced by waist circumference.

**Conclusions:**

Adipocytokine levels may be independent predictors of endothelial dysfunction in the peripheral circulation of healthy subjects, providing a pathophysiological link between inflammation from adipose tissue and early vascular alterations.

## Background

Endothelium integrity is essential in the maintenance of vascular homeostasis, and its dysfunction, identified as a reduced vasodilatory response to endothelium-dependent stimuli, predisposes to the development of early atherosclerotic lesions [1]. Therefore, endothelial dysfunction is recognized as at least a marker, if not an independent risk factor for cardiovascular events [1,2].

Although the term “dysfunction” is mainly used in reference to a loss of nitric oxide (NO) bioavailability, it also reflects increased production of vasoconstrictor agents and disturbed regulation of inflammation, thrombosis and cell growth in the vascular wall [3]. All these phenomena may identify a pathogenic link with several chronic diseases, such as type 2 diabetes and essential hypertension, either characterized by an early defect in endothelial function and a certain degree of subclinical inflammation [4].

Some inflammatory mediators originating from the adipose tissue can lead to local insulin resistance with an impaired inhibitory effect of insulin on the release of free fatty acids (FFAs) and endothelial dysfunction, thus assuming a special meaning in promoting early arterial damage. Among these, tumor necrosis factor-α (TNFα) has been linked with reduced insulin sensitivity and decreased endothelial NO synthase expression in cultured endothelial cells [5], and interleukin −6 (IL-6) is implicated in endothelial dysfunction and vascular inflammation through several mechanisms, including induced expression of monocyte chemotactic protein-1 (MCP-1) [6]. More recently, retinol binding protein-4 (RBP4), an adipocytokine linked to insulin resistant states in mouse animal models and in humans [7,8] and predicting the development of type 2 diabetes [9], has been associated to endothelial dysfunction in new-onset type 2 diabetes [10]. Circulating levels of RBP4 are inversely related with brachial artery flow-mediated dilation (FMD) in type 2 diabetic individuals [11], and RBP4 levels are also increased in other clinical conditions characterized by increased cardiovascular risk and a certain degree of insulin resistance, such as essential hypertension [12] and metabolic syndrome [13].

Another adipocytokine potentially influencing endothelial function is resistin, whose plasma levels correlate with markers of inflammation and are predictive of coronary atherosclerosis in humans [14]. More recently, resistin has been shown to induce Intercellular Adhesion Molecule 1 (ICAM-1) and Vascular Cell Adhesion Molecule 1 (VCAM-1) expression in endothelial cells, thus promoting monocyte recruitment and inflammatory responses [15].

No study has so far evaluated the relationship of these adipocytokines with endothelial function in drug-naïve, newly diagnosed hypertensive subjects without organ damage and in normotensive matched individuals; the present study has been aimed to address this specific issue.

## Subjects and methods

### Patients

92 untreated neo-diagnosed, drug-naïve essential hypertensive patients (HT) were consecutively recruited among those referring to the Hypertension Outpatient Clinic, Department of Internal Medicine of the University of Pisa. Exclusion criteria were diabetes mellitus (fasting plasma glucose >126 mg/dl or pharmacologic therapy), smoking history (more than 5 cigarettes per day), severe hypercholesterolemia (total cholesterol greater than 6.2 mmol/L), cardiac and/or cerebral ischemic vascular disease, impaired renal function, heart failure, conductance disturbances, acute inflammatory or other major diseases. Target organ damage was excluded according to current guidelines [16]. Sixty-six non diabetic normotensive subjects without family history of essential hypertension and blood pressure (BP) values below 140 and 90 mmHg, recruited on a volunteer basis, served as controls (NT). The local Ethical Committee approved the protocol, and all participants gave their written informed consent.

### Experimental procedures

All participants were asked not to eat, and to avoid caffeine-containing beverages, alcohol, strenuous exercise and smoking for the 12 h prior to the experiment. Measurements were performed in the morning with participants supine and at rest in a quiet air-conditioned room (22-24°C). Before starting the study, a blood sample was drawn from an antecubital vein. After collection, the blood was rapidly centrifuged at 1500 rpm for 5 min to separate serum and plasma from clot-containing blood cells, storing samples at −70 C until analysis.

### Vascular function

Endothelium-dependent vasodilation was assessed as flow-mediated dilation (FMD) of the brachial artery [17], as previously described [18]. Briefly, after 1 min of baseline acquisition, the cuff was inflated for 5 min and then deflated to induce reactive hyperemia (RH). Endothelium-independent dilation was obtained by administration of 25 μg sublingual glyceryl trinitrate (GTN). Brachial artery diameter was measured on acquired frames by a computerized edge detection system (FMD studio, Quipu s.r.l, Pisa, Italy) [19,20]. FMD and response to GTN were calculated as the maximum percent increase in diameter above baseline after RH and GTN administration, respectively. Blood flow volume was calculated by multiplying Doppler flow velocity (corrected for the angle), heart rate, and vessel cross-sectional area (*πr*^2^) at baseline and within 15 s after cuff release to calculate RH (as percent increase in blood flow).

### Biochemical determinations

Glucose and serum lipids were assayed by standard enzymatic methods. Serum creatinine was measured by the modified Jaffe method. High-sensitive C-reactive protein (hsCRP) was measured using a high-sensitive (low detection limit, 0.3 mg/l) immunoassay (N High Sensitivity CRP, Dade Behring, Marburg, Germany). Estimated glomerular filtration rate (eGFR) was calculated using the recently developed Chronic Kidney Disease Epidemiology Collaboration (CKD-EPI) equation [21].

E-selectin was measured by ELISA (R&D Systems, Abingdon, UK), with intra- and inter-assay coefficients of variation of <5% and <10% respectively. Oxidative stress was evaluated by measurement of plasma malondialdehyde (MDA) concentrations, as previously described [22], with an intra- and inter-assay coefficient of variation (CV) of 6% and 11%, respectively.

Plasma concentrations of RBP4 were determined by a commercially available ELISA (R&D System GmbH, Wiesbaden-Nordenstadt, Germany). The intraassay CV was 4.7% and the interassay CV was 5.0%. Results were validated by performing Western blot analysis in a subset of ten controls and ten hypertensive patients. Briefly, sera were diluted 1:20 in SDS-PAGE buffer and heated at 100°C for 5 min. Samples (5 μl) and molecular weight markers were electrophoresed on 15% Trys-Glycine SDS-PAGE gels and transferred to PVDF membrane (Millipore, Billerica, MA, USA). After a blocking step using BSA 3% in TTBS (TBS and Tween-20 0.05%) for 1 h at room temperature, blots were washed three times in TTBS and incubated overnight at 4°C with primary antibody anti-human RBP4 (ab57620 Abcam, Cambridge, MA, USA) diluted 1:400. The bands detection was performed incubating the blot with horseradish-peroxidase-conjugated secondary antibody (AP308P Chemicon, Temecula, CA, USA) diluted 1:4000 for 1 h at room temperature, followed by enzymatic chemiluminescence kit (34075 Pierce Biotechnology, Rockford, IL, USA).

Plasma levels of resistin and TNFα were measured by ELISA (R&D System, Minneapolis MN, USA; intraassay CV 3.8% and interassay CV 6% for resistin and 10.3% and 6.0% for TNFα, respectively).

### Statistical analysis

Statistical analysis was performed using NCSS 2004 (NCSS, Kaysville, UT, USA). The results were expressed as mean ± SD or median (range). Differences among groups were analyzed using Student *t* test or Mann–Whitney *U*-test. Continuous variables were compared by Pearson's or Spearman's rank correlation coefficient. Multiple linear regression was applied to build a model to identify the determinants of FMD. Non-normally distributed variables were log-transformed for this analysis. A value of p <0.05 was considered significant.

## Results

Clinical characteristics of the two groups of subjects are reported in Table 1. HT and NT did not differ for clinical characteristics with the exception of BP values and triglycerides levels; even hsCRP did not differ between the two groups.

**Table 1:** Clinical characteristics of the studied subjects. Data are expressed as mean ± SD or median (range)

	**NT (n = 66)**	**HT (n = 92)**
Age (years)	47.1 ± 10.7	48.9 ± 9.9
Gender (male/female)	46/20	65/27
Body Mass Index (kg/m^2^)	26.7 ± 4.0	26.5 ± 3.9
Waist circumference (cm)	94.0 ± 11.1	94.7 ± 11.5
Smokers (yes/no)	11/55	21/71
Systolic BP (mmHg)	131.6 ± 9.0	150.9 ± 11.5*
Diastolic BP (mmHg)	81.5 ± 6.5	92.9 ± 8.5*
Fasting glucose (mg/dl)	93.6 ± 13.1	94.1 ± 12.8
Total cholesterol (mg/dl)	205.5 ± 32.1	203.3 ± 37.8
HDL cholesterol (mg/dl)	49.9 ± 14.4	52.0 ± 15.8
LDL cholesterol (mg/dl)	127.7 ± 36.7	127.8 ± 34.3
Triglycerides (mg/dl)	93 (68–147)	128 (92–174)*
Creatinine (mg/dl)	0.89 ± 0.49	0.93 ± 0.63
eGFR (ml/min/1.73 m^2^)	87.8 ± 11.9	84.2 ± 16.7
hsCRP (mg/l)	1.4 (0.4-3.6)	2.8 (1.3-5.9)

*p <0.05 vs normotensive patients.

HDL: High density lipoproteins; LDL: low density lipoproteins; eGFR: estimated glomerular filtration rate.

**Figure 1:**
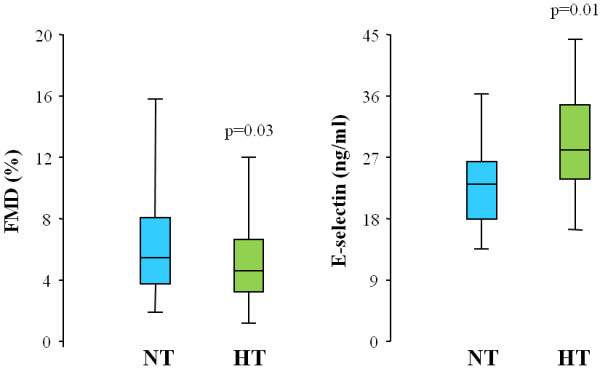
**Flow mediated dilation and plasma E-selectin levels in the two study groups.** Box-plots show flow mediated dilation (FMD, expressed as percent of increase in brachial artery diameter above baseline after reactive hyperemia), and E-selectin levels in normotensive subjects (NT, blue plots) and essential hypertensive patients (HT, green plots).

As expected, FMD was significantly lower in HT as compared to NT (Figure 1), while response to GTN (7.5 ± 3.7% *vs* 7.9 ± 3.4%), diameter (0.43 ± 0.09 *vs* 0.41 ± 1.02 cm) and peak shear rate (6.33 ± 2.78 *vs* 6.28 ± 2.89 s^-1^) were similar. In agreement with the reduced FMD, HT also showed higher levels of the endothelial-specific protein E-selectin (Figure 1), while MDA levels did not differ between HT and NT (2.39 ± 1.26 and 2.08 ± 1.17 nmol/ml, respectively). No relationship was found between hsCRP and either FMD and MDA levels in the two groups.

**Figure 2:**
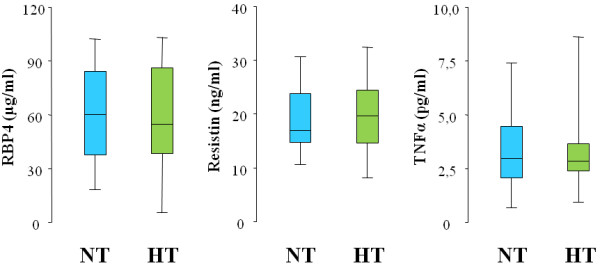
**Retinol binding protein-4, resistin and TNFα levels in the two study groups.** Box-plots show retinol binding protein-4 (RBP4), resistin and TNFα levels in normotensive subjects (NT, blue plots) and essential hypertensive patients (HT, green plots).

The different behaviour of FMD and E-selectin in HT *vs* NT individuals was not paralleled by a concomitant variation of adipocytokine levels: in fact, RBP4, resistin and TNFα did not differ between the two groups (Figure 2). This was true even when we compared smokers with non smokers (data not shown).

RBP4 levels were significantly related to resistin in the overall population (r = 0.46; p <0.0001) and in NT (r = 0.89; p <0.0001), but not in HT (r = −0.01; p = 0.97). No relationships were found among TNFα and adipocytokines levels, excluding with resistin in HT (r = −0.32; p = 0.02).

Analyzing linear correlations between RBP4, resistin, TNFα and the clinical parameters in NT, RBP4 was related to diastolic BP (r = 0.25; p = 0.04) and MDA (r = 0.28; p = 0.03), but not to BMI and-or waist circumference. Resistin was related to diastolic BP (r = 0.32; p = 0.01), log transformed triglycerides (r = 0.34; p = 0.01) and waist circumference (r = 0.24; p = 0.04), but not with BMI. In HT, the only significant relationship was between resistin and plasma glucose (r = 0.33, p = 0.01). TNFα was not related with any clinical parameter, neither in NT and HT individuals. No relationship was found between any adipocytokine and eGFR value, either in NT and HT individuals.

We then explored the putative relationship between cytokine levels and endothelial function. Either E-selectin levels (a reliable proxy of endothelial activation) or response to GTN did not correlate with FMD and RBP4 or resistin. The only significant correlates of E-selectin were systolic and diastolic BP in the overall population (r = 0.31 and 0.29, respectively; both p <0.001).

**Figure 3:**
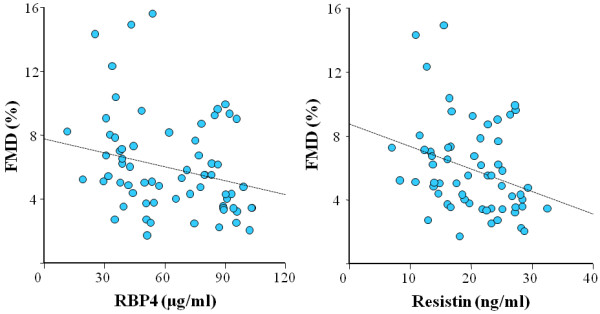
**Correlation between flow mediated dilation and retinol binding protein-4 and resistin levels in normotensive subjects.** Scatter-plots of the correlation between flow mediated dilation (FMD, percent increase in brachial artery diameter) and retinol binding protein-4 (RBP4, r = −0.25; p = 0.04, left) and resistin (r = −0.29; p = 0.03, right) levels in normotensive individuals.

RBP4 (r = −0.25; p = 0.04) and resistin (r = −0.29; p = 0.03) were related to FMD in NT (Figure 3), but not in HT (r = −0.03; p = 0.81 and r = −0.10; p = 0.46, respectively); other significant correlates of FMD in NT were brachial artery diameter (r = −0.60; p <0.001), age (r = −0.29; p = 0.02), BMI (r = −0.33; p = 0.008), waist circumference (r = −0.35; p = 0.006) and log transformed triglycerides (r = −0.29; p = 0.02).

Multivariate analysis performed in NT, including confounders (age, BMI, diastolic BP, log transformed triglycerides, MDA) and RBP4, showed that only BMI remained significantly related to FMD (Table 2). Similar results were obtained when BMI was replaced by waist circumference. Multivariate analysis performed in NT, including confounders (age, BMI, diastolic BP, log transformed triglycerides, resistin) showed that BMI, age and resistin were significantly related to FMD (Table 2). When BMI was replaced by waist circumference in the model, only age remained significantly related to FMD.

**Table 2:** Adipocytokines and clinical and vascular factors affecting flow mediated dilation in the normotensive individuals- multivariate analysis

**Model 1**	**r**^**2**^ **= 0.25**	**Model 2**	**r**^**2**^ **= 0.29**
**Factor**	**p value**	**Factor**	**p value**
Age	0.06	Age	0.14
BMI	0.02	Waist	0.04
Diastolic BP	0.75	Diastolic BP	0.56
Ln (triglycerides)	0.64	Ln (triglycerides)	0.55
MDA	0.81	MDA	0.75
RBP4	0.07	RBP4	0.08
**Model 3**	**r**^**2**^ **= 0.29**	**Model 4**	**r**^**2**^ **= 0.15**
**Factor**	**p value**	**Factor**	**p value**
Age	0.03	Age	0.03
BMI	0.01	Waist	0.27
Diastolic BP	0.85	Diastolic BP	0.71
Ln (triglycerides)	0.16	Ln (triglycerides)	0.29
Resistin	0.03	Resistin	0.29

BMI: body mass index; BP: blood pressure; Ln: log-transformed; MDA: malondialdehyde.

## Discussion

### Relationship between adipocytokines and endothelial function

RBP4 has been previously related to insulin resistance and clinical markers of increased cardiovascular risk such as intima-media thickness (IMT), not only in high-risk individuals including hypertensive and type 2 diabetic and hypertensive patients [10,12], but also in the general population [23]. Moreover, RBP4 levels have been related with *in vivo* evaluation of endothelial function in patients with type 2 diabetes [11], but such relation has not been explored so far in essential hypertension. Reports dealing with the role of resistin during the course of hypertension are more contradictory: resistin is able to activate human endothelial cells, increasing the expression of adhesion molecules and ET-1 release [15,24]; its plasma levels are inconstantly elevated in patients with essential hypertension [12,25], and are not in elderly people [26].

We show here for the first time that *i*) in neodiagnosed, drug-naïve hypertensive individuals, neither RBP4 nor resistin are related with endothelial dysfunction; *ii*), *vice versa*, in healthy (normotensive non diabetic and non obese) individuals, even in the presence of RBP4 and resistin levels within the normal range, a strict relationship exists between these adipocytokines and endothelial function.

### Differences between normotensive and hypertensive individuals

In our hands, RBP4 levels did not differ between hypertensive and normotensive individuals; however, any putative influence of dietary intake on RBP4 levels [27] of our study participants might reasonably be excluded from personal history on life habits. This finding is partially at odd with our previous published data [12], which were, however, obtained in a cohort of women, while the male gender was largely prevalent in both groups of the present study population. A recent report suggests a role of smoking in influencing RBP4 concentrations in Chinese healthy individuals [28]: in our Caucasian population (where smokers were about one fifth), this observation was not confirmed.

We were also unable to find differences in resistin levels between hypertensive and normotensive subjects, as already reported in women [12]. A possible explanation could be that in both studies we evaluated only neo-diagnosed patients, with a presumably short duration of the disease and no clinically evident organ damage, while resistin is elevated in patients with inflammatory vascular damage [29] and advanced atherosclerotic disease [30,31]; another possibility could be that our patients had all a preserved kidney function, while a reduced GFR has been linked to increased resistin levels in a large cohort of community-dwelling subjects [32].

### Relationship between FMD, MDA and adipocytokines

Concerning endothelial function in the peripheral microcirculation, brachial artery FMD is reduced in hypertensive as compared to normotensive subjects, confirming previous results [18]. Since endothelium-independent vasodilation and peak shear rate were similar, these results indicate the presence of endothelial dysfunction; accordingly, HT showed also increased levels of E-selectin, a marker of endothelial activation [33]. At variance with that reported in patients with type 2 diabetes [10], RBP4 levels were not related to FMD in hypertensive individuals carrying endothelial dysfunction; similarly, E-selectin did not correlate with RBP4 in these patients. The lack of this relationship might be that endothelial dysfunction might recognize different mechanisms in diabetes and essential hypertension [34]; an alternative explanation could be the almost superimposable RBP4 concentrations between the two groups, making impossible to pick up small differences in the correlations. However, our results might indirectly confirm the scarce role of RBP4 as early marker of subclinical vascular impairment, as recently documented [35]. Another potential explanation for the lack of these correlations may be due to the fact that none of these subjects showed any clinical sign of autonomic neuropathy, another condition influencing plasma adipocytokine concentrations, for example in type 2 diabetes [36].

Quite surprisingly, in healthy normotensive subjects RBP4, but not E-selectin, was significantly related to FMD and MDA, suggesting a specific role for this adipokine in marking endothelial dysfunction and oxidative stress, but not endothelial activation. However, multivariate analysis revealed that, even in slim healthy individuals, indexes of adiposity (BMI or waist circumference) remain the only independent determinants of FMD, again underlining the possible role of RBP4 as marker of early endothelial dysfunction, even in the absence of excess of fat depots or other CV risk factors. The possibility that arterial diameter influences the relationship between FMD and BMI is plausible; however, since brachial artery diameter is highly related with FMD, this hypothesis cannot be formally tested, because its inclusion in the model might cause statistical multicollinearity.

### Role of resistin and TNFÎ±

The relationship among resistin, hypertension and endothelial function is even more complex. A direct influence of resistin in promoting endothelial activation and influencing endothelial function has been so far described only in experimental models [24,37,38], even though pharmacological interventions in nondiabetic hypertensive individuals might reduce resistin levels in parallel with an improvement of endothelial function [39,40]. Here, we show for the first time that, as for RBP4, no relationship is present between resistin levels and *in vivo* repeatable assessment of endothelial function in untreated essential hypertension. However, the relationship between resistin and endothelial function in healthy normotensive individuals without family history of hypertension assumes a special meaning, since resistin levels remained significant correlates of FMD also in the multivariate analysis, independently of BMI and waist circumference. These results could be also related to the recent report that resistin, as already shown for reduced FMD [41], predicts the development of hypertension in healthy women [42].

The lack of correlation between TNFα levels and FMD might surprise, given the relevant contribution of this cytokine to the pathogenesis of endothelial dysfunction [43]. This finding could be due to the fact that TNFα mirrors the degree of subclinical inflammation, almost absent in our study population, as shown by hsCRP levels, relatively low either in HT and NT subjects and not related with any marker of endothelial function.

## Conclusions

Our data suggest that RBP4 and resistin might mark an early vascular dysfunction not detected by common vascular indicators like E-selectin, which is a likely indicator of a more advanced degree of endothelial impairment. The main limitations of this study include the small sample size, that implies to consider the results as a simple clinical observation deserving further confirmation in larger populations; however, these preliminary observations might provide a pathophysiological link between inflammation from adipose tissue and early vascular alterations.

## Abbreviations

CV: Coefficient of variation; eGFR: Estimated glomerular filtration rate; FMD: Flow-mediated dilation; FFAs: Free fatty acids; GTN: Glyceryl trinitrate; hsCRP: High-sensitive C-reactive protein; HT: Hypertensive patients; ICAM-1: Intercellular Adhesion Molecule 1; IL-6: Interleukin-6; IMT: Intima-media thickness; MCP-1: Monocyte chemotactic protein-1; MDA: Malondialdehyde; NO: Nitric oxide; NT: Normotensive controls; RH: Reactive hyperemia; RBP4: Retinol binding protein-4; TNFα: Tumor necrosis factor-α; VCAM-1: Vascular Cell Adhesion Protein.

## Competing interests

L. Ghiadoni is councilor of Quipu s.r.l, Pisa, Italy.

## Authors’ contributions

AS designed the study and wrote the paper; FS and RMB recruited the patients and performed the studies; ES and ED performed the lab work; ST revised the paper; LG designed the study, analyzed data and contributed to write the paper. All authors read and approved the final manuscript.
